# Improving disaster risk reduction capacity of District Civil Protection Units in managing veld fires: A case of Mangwe District in Matabeleland South Province, Zimbabwe

**DOI:** 10.4102/jamba.v7i1.143

**Published:** 2015-05-25

**Authors:** Ernest Dube

**Affiliations:** 1 Department of Development Studies, Midlands State University, Zimbabwe

## Abstract

This article analysed disaster risk reduction capacity of District Civil Protection Units (DCPUs) in managing veld fires in Mangwe District of Matabeleland South Province, Zimbabwe. Veld fires have resulted in unnecessary material, environmental and economic losses. Communities’ livelihoods and property have been destroyed, and the natural environment depleted. The research sought to improve disaster risk reduction capacity of DCPUs in managing veld fires, through new intervention strategies and a new model. The objectives of the study were to investigate the main causes of veld fires; to analyse their impacts; to examine the effectiveness of the current intervention strategies; and to identify challenges in implementing these interventions. Furthermore, the study sought to recommend new possible intervention strategies. This mainly qualitative study employed self-administered questionnaires, interviews and focus-group discussions. Questionnaires were used to investigate members of the DCPU's ideas, views and experiences, interviews solicited perceptions of community leaders and their subjects, whilst focus-group discussions assisted with information from members of the District Civil Protection Planning Committee. Veld fires in the district are mainly caused by human activities, and they are prevalent during the months of September and October. They affect livelihoods and the natural environment the most. This study found that DCPUs are not prepared to manage veld fires and therefore recommended new strategies and adoption of the community-based disaster risk reduction model. The new strategies include involving community leaders and members of the communities in DCPUs; regular training and workshops to members of DCPUs on veld fire management; creation of fire protection associations; regular campaigns and rehearsal of emergency drills by the DCPU personnel; the introduction of competitions and incentives in veld fire management; vigorous public education on the erection of proper fireguards around homes, cattle pens, crop fields and vegetable gardens; and the imposition of stiffer penalties for carelessly or deliberately causing veld fires. Policy-makers, governments and stakeholders would benefit from the new intervention strategies. The community-based disaster risk reduction model would benefit researchers and disaster risk reduction practitioners.

## Introduction

**Figure 1:**
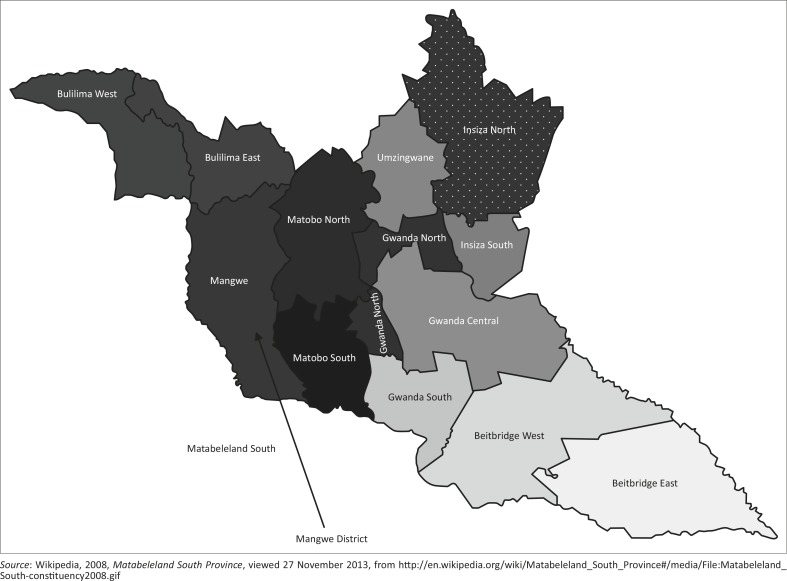
Position of Mangwe District in Matabeleland South Province, Zimbabwe.

Disaster risk reduction capacity entails measures employed to deal with any hazard, and veld fires are no exception. Veld fires are increasingly occurring on a regular basis in Mangwe District in Matabeleland South Province in Zimbabwe. The district shares its boundary with the Republic of Botswana in the west, Matobo district in the east and Bulilima district in the north (Figure 1). Its population is estimated at 66 218 based on the 2012 census (Zimstat 2012).

Most of the veld fires in Mangwe District are seemingly caused by human activities, such as uncontrolled and indiscriminate burning of vegetation and by sparks emanating from passing trains. As observed by Mkhwananzi (2007), human beings have contributed to most of the veld fires, and it is believed that they are responsible for 95% of all forest and veld fires. Nkomo and Sassi (2009) also note that veld fires outbreaks in southern Africa have resulted mainly from human activities such as land clearing, hunting, pasture management and crop production. During land clearing activities, some farmers start destructive fires, which have had negative effects by damaging the environment. Veld fires are therefore an environmental problem, with human activity at the epicentre. In addition, people's wealth, such as livestock, crops, pastures, wild fruit and game, are put in jeopardy by devastating veld fires. Veld fires in Zimbabwe destroyed 950 905 hectares of farming land in 2009; 1 152 413 in 2010; 713 770 in 2011 and 1 320 325 in 2012 (Environmental Management Agency [EMA] 2011), thereby threatening food security of the country. Against this background disaster preparedness by all stakeholders, including the District Civil Protection Units, in managing veld fires is of paramount importance.

Disaster risk reduction measures for veld fires therefore involve focusing on early warning systems and ways to improve readiness of key actors to respond appropriately to fires as they occur. They also involve the development of early warning mechanisms, plans for evacuation, the education and training of officials and people at risk, the establishment of policies, standards, organisational arrangements and operational plans, and securing resources for training intervention teams in both pre-disaster and post-disaster periods (Kotze & Holloway 1996). Kent (1994:11) concurs with this statement by asserting that ‘preparedness planning improves the response to the effects of a disaster by organising the delivery of timely and effective rescue, relief and assistance’. Such plans must be supported by enabling legislation (Kent 1994).

The main objective of the study was to improve disaster preparedness of District Civil Protection Units (DCPUs) in managing veld fires. The sub-objectives were to:

- investigate the main causes of veld fires
- analyse their impacts
- examine the effectiveness of the current intervention strategies
- identify challenges in implementing these interventions
- recommend new possible intervention strategies.

It is important, therefore, that DCPUs bring disaster risk reduction measures to the fore, evaluate them, and map out strategies so that they are able to deal with veld fire hazards in order to prevent the occurrence of any disaster.

### Problem statement

Veld fires are increasingly occurring on a regular basis in Mangwe District in Matabeleland South Province, Zimbabwe. The veld fires have resulted in unnecessary material, environmental and economic losses. Communities’ livelihoods and property have been destroyed, and the natural environment depleted. These veld fires have been causing massive destruction in the district, despite the existence of the DCPU, whose mandate is to deal with all forms of disasters occurring in the district. The DCPU in Mangwe has always tried to act upon this kind of hazard and the resultant disaster. However, it seems the unit is not well capacitated to manage the phenomenon, as evidenced by the continued losses of property, livelihoods and the environmental degradation. The aim of this study was therefore to help improve disaster risk reduction of the DCPUs in managing veld fires, with specific focus on Mangwe District. It sought to investigate the main causes of veld fires; to analyse their impacts; to examine the effectiveness of the current intervention strategies; and to identify challenges in implementing these interventions. Policy-makers, governments and stakeholders would benefit from the new intervention strategies. Furthermore, the study attempts to recommend new possible intervention strategies for managing veld fires. Lastly, a new model for disaster risk reduction, namely the community-based disaster risk reduction model, is presented. Disaster risk reduction practitioners and other researchers are expected to benefit from this model.

### Literature review

Veld fires have had varying negative impacts on humans, livelihoods and the natural environment, leading to severe losses. In the context of human development, loss of livelihoods should also be taken into account. With regard to environmental losses, veld fires have destroyed pastures and forced animals to migrate elsewhere, posing a serious threat to wildlife management. Veld fires have also resulted in a decline in veld conditions, as well as in the degradation of the ecosystem (Everson, George & Schulze 1989). Although the effects of veld fires on the natural environment may seem temporary, there are long-term impacts on biodiversity that may be irreversible. Veld fires increase air pollution, thereby reducing the quality of air that people breathe. This leads to health problems and also contributes to the depletion of the ozone layer, leading to climate change in the form of global warming. Disaster risk reduction plans to deal with this phenomenon should therefore not be ignored by governments and disaster risk reduction managers. A vibrant disaster risk reduction plan is a prerequisite.

According to the United Nations Development Programme (1992), there are nine major components involved in a disaster risk reduction preparedness plan, which provides a framework upon which a national disaster preparedness strategy can be developed. Just like any kind of disaster, veld fires require a high standard of disaster risk reduction preparedness. A disaster risk reduction preparedness plan ensures that communities are in a good position to deal with disasters and reduce their impacts. Masellis, Ferrara and Gunn (1999) define preparedness as:

the aggregate of measures to be taken in view of disasters, consisting of plans and action programmes designed to minimise loss of life and damage, to organise and facilitate effective rescue and relief, and to rehabilitate after disaster. (p. 67)

Preparedness also requires the necessary legislation and means to cope. Kent (1994:11) points out that disaster preparedness ‘involves forecasting and taking precautionary measures prior to an imminent threat when advance warnings are possible’.

## Disaster preparedness framework

The disaster preparedness framework (Table 1) is used to present the nine elements of the disaster preparedness plan. The plan contains the activities that should be performed in preparation for an impending disaster. The elements of the plan can also be adopted when preparing for veld fires. Although an implementation sequence for these activities is suggested (Table 1), some activities may be undertaken simultaneously, or even in the reverse order.

**Table 1:** The disaster preparedness framework.

**Vulnerability assessment**	**Planning**	**Institutional framework**
Information systems	Resource base	Warning systems
Response mechanisms	Public education and training	Rehearsals/emergency drills

*Source*: United Nations Development Programme (UNDP), 1992, *An overview of disaster management*, 2nd edn, Disaster Management Training Programme, UNDP/UNDRO, New York and Kent, R., 1994, *Disaster preparedness*, 2nd edn., Disaster Management Training Programme, UNDP, p. 16, viewed 10 October 2013, from http://www.pacificdisaster.net/pdnadmin/data/original/dmtp_07_disaster_preparedness_8.pdf

In the preparedness plan, *vulnerabilities assessment* assists in providing help that will support the efforts of the affected people to attain social and economic development. Cannon (2003:11) contends that vulnerability assessment was ‘designed principally for NGOs, to help them consider when and how to respond to a disaster by understanding what impact interventions will have on capacities and vulnerabilities’. *Planning* is the theme of the whole disaster preparedness exercise. Planning for readiness to deal with disasters includes working out agreements between people or agencies, as to who will provide services in an emergency to ensure an effective, coordinated response (Kent 1994). An *institutional framework* entails an existing and coordinated structure mandated with disaster risk management activities. Therefore, there is no need for creating new organisations, but use should be made of the established structures and systems. This is the case with Zimbabwe, where existing governmental structures and systems have been adopted. *Information systems* are also a vital component of the disaster preparedness plan. Disasters can happen because people vulnerable to them simply do not know how to get out of harm's way or to take protective measures (United Nations Development Programme [UNDP] 1992:19). Information dissemination concerning disasters puts communities in a good position to take proper measures. A good example is information on fire danger rating, which is usually disseminated by the Meteorological Department in Zimbabwe. A strong *resource base* is also a vital component of the disaster preparedness framework. Specific arrangements should be established whereby each party to written agreements can secure goods and services as required. *Warning systems* include having functioning communications systems, such as telephones, police radios and telexes, which are vital in times of a major disaster. *Response mechanisms* for disasters entail a number of responses, depending upon the nature of the hazard. Some of the broader categories of responses (UNDP 1992) are:

- evacuation procedure
- search and rescue
- security of affected areas
- assessment teams
- activating special installations (such as emergency hospital facilities)
- activating distribution systems
- preparing emergency reception centres and shelters.

*Public education and training* is an intervention that provides people with information needed to reduce the effects of disasters. Coburn, Spence and Pomonis (1994a:37) emphasise that ‘disaster awareness can be promoted through national days or months dedicated to hazard related exercises’. In this case, veld fire campaigns in Zimbabwe can be undertaken every year, around the month of September, to increase awareness. Awareness of the risk by the public in general and perception of how it compares to other risks will determine society's attitudes about reducing it (Coburn *et al*. 1994b:9). In the education of disaster preparedness, it is important to give people the information relevant to their level of participation in the community (Erdik 1991). Ward (1991:136) emphasises that training must be appropriate to the level at which it is conducted. Potential disaster victims need to be shown what they can do to help themselves when a disaster strike, relief workers need to be trained to help others, and community leaders must be shown how to prepare their communities and so on (Ward 1991:23).

*Rehearsals/emergency drills* as a risk reduction strategy cannot portray the full dynamics and chaos of a disaster relief operation. However, that cannot be an excuse for avoiding the need to rehearse the disaster preparedness plan. According to the National Fire Protection Association (National Fire Protection Association [NFPA] 2000), the primary objective of the fire drill is an orderly evacuation: ‘in the conduct of drills, emphasis shall be placed on orderly evacuation rather than on speed’. Rehearsals usually reemphasise points made in separate training programmes, and test the system as a whole. They invariably expose gaps that otherwise might be overlooked.

## Disaster risk reduction legal framework

Although the components of the disaster preparedness framework presented above are a useful aid in managing disasters, the framework does not incorporate legislation. Legislation is vital as it gives direction to the management of disasters in a country, and therefore it must form part of the preparedness plan. This is supported by InterWorks (1998:10), whose view is that to exercise a disaster preparedness strategy, agencies must be supported by policies, legislation, agreements as well as resources.

The main legislation governing disaster preparedness in Zimbabwe is the *Civil Protection Act* Chapter 10:06 of 1989 (Government of Zimbabwe 2001). This Act is the successor of the *Civil Defence Act* of 1982. According to this Act, it is national policy for civil protection that every citizen of the country should assist where possible to avert or limit the adverse effects of disasters. This legislation resulted in the creation of the Department of Civil Protection (Bongo *et al*. 2013). The Department is currently housed within the Ministry of Local Government, Rural and Urban Development as the implementing body of the national government-initiated disaster preparedness and mitigation programmes (Chikoto 2004). The Department is responsible for coordinating all national response efforts (UNISDR 2005). Its primary functions include preparing for, preventing where possible, and mitigating the effects of disasters.

The *Civil Protection Act* Chapter 10:06 has its shortfalls. Part I, Section 2 of the Act assigns the administration of the Act and coordination of disaster activities in the country to the Minister of Local Government, Rural and Urban Development. Considering that the minister responsible for this portfolio has other issues not connected to disaster risk reduction to focus on, the minister may not be effective with regard to disaster risk reduction issues. According to Part VI, Section 20(1) of the Act, volunteers who want ‘to serve in a civil protection organisation for the protection area’, should first apply to the area civil protection officer for the area concerned. This may be a long procedure working against efficiency, considering that some disasters are the rapid onset type.

### Structural model of the Zimbabwe civil protection system

**Figure 2:**
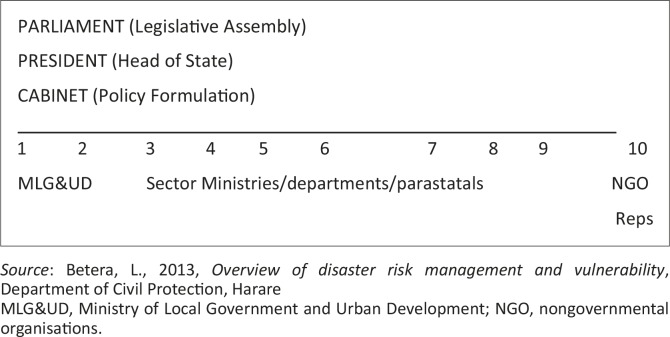
Zimbabwe civil protection system.

The civil protection system in Zimbabwe (Figure 2) uses the existing government, private and nongovernmental organisations whose regular activities contain elements of preparedness, prevention and community development. These organisations are adopted structurally, materially and technically so that they can speedily shift from their regular activities to undertake preventive, relief and rehabilitation measures in times of disasters in terms of intensity only without drifting from their usual operational principles. The hierarchy of the Zimbabwe civil protection system consists of the legislative assembly, represented by Parliament; the head of state, who is the President; and the Cabinet, which is responsible for policy formulation. Under this hierarchy is the Ministry of Local Government and Urban Development, which is mandated with the coordination role during disasters and emergencies in Zimbabwe through the Department of Civil Protection (DCP). The Ministry works with sector ministries and nongovernmental organisations (NGOs) in dealing with any type of disasters.

There are challenges in implementation of the structure of the Zimbabwe civil protection system. The structure puts the President under Parliament. However, the *Civil Protection Act* empowers the President to declare a state of disaster in the country (Government of Zimbabwe 2001). This is a function that the President performs without consulting Parliament. Parliament therefore is rendered less effective in dealing with disaster risk reduction issues.

### Civil protection organisation in Zimbabwe

The *Civil Protection Act*, Chapter 10:06, creates the operational structure of the Civil Protection Organisation, which operates at national, provincial and district level. This means that the Act establishes a Civil Protection Unit (CPU) at each level, whose functions are coordinated by respective Civil Protection Planning Committees (CPPCs). At the national level, the CPPC is chaired by the minister responsible for local government. At provincial level, the committee is chaired by the Provincial Administrator, whilst at district level the committee is chaired by the District Administrator. Other members, who are part of the CPPC at all three levels, are drawn from different government departments, including the police, defence forces, Department of Social Welfare and local hospitals, as well as NGOs, such as the Red Cross Society, Save the Children Zimbabwe, and the International Organization for Migration (IOM).

Apart from the *Civil Protection Act*, Chapter 10:06, there are other Acts that underpin disaster risk reduction activities and management in Zimbabwe. The principal ones are the *Environmental Management Act*, Chapter 20:27 (Government of Zimbabwe 2002a), the *Rural District Councils Act*, Chapter 29:13 (Government of Zimbabwe 2002b) and the *Forestry Act*, Chapter 19:09 (Government of Zimbabwe 2002c). The *Environmental Management Act* provides for the establishment of the EMA, which it empowers to be the environmental management watchdog in Zimbabwe. Part IX of the Act (Government of Zimbabwe 2002a) deals with environmental quality standards, which has a bearing on disaster preparedness and prevention. According to Statutory Instrument Number 7 of 2007, as read with the Act, no person is allowed to light a fire outside residential and commercial premises during the period August to October (Zimbabwe National Parks and Wildlife Authority 2014). However, the enforcement of this Act in Mangwe District is not effective. The EMA as the environmental watchdog is not well versed in and confident with law enforcement. On the other hand the police, who are well versed in law enforcement, are not effective either. This is so because they do not focus all their energies in enforcing this Act only, but deal with all legislation.

The *Rural District Councils Act* provides for the administration of Rural District Councils (RDCs) and gives them custodianship of natural resources. Part X of the Act (Government of Zimbabwe 2002b) stipulates the powers and duty of RDCs. Section 71 empowers the RDCs to undertake various issues, as listed in the first schedule. Most of these have a bearing on disaster preparedness and conservation of the environment. For example, RDCs are mandated to take measures to control bush fires, regulate farming and pollution. The Act also empowers the RDCs to make by-laws and to conserve natural resources. According to the Act, the by-laws can be enacted with regards to such issues as amenities and facilities, water and its pollution, effluent and solid waste management and removal of vegetation.

The *Forestry Act* (Government of Zimbabwe 2002c) establishes a Forestry Commission for the administration, control and management of state forests. The main purpose of the Commission is to oversee the protection of private forests, trees and forest produce; and to regulate and control the burning of vegetation. All this is done in order to provide for the conservation of the fauna and flora, which is usually depleted either through uncontrolled cutting down of plants or through indiscriminate and uncontrolled burning. Part VIII of the Act deals with the control of fires and burning of vegetation. Section 67(1) of the Act mentions that ‘no person shall burn growing or standing vegetation on any land’ (Government of Zimbabwe 2002c). The Act compliments the *Environmental Management Act*, Chapter 20:27, and the *Rural District Councils Act*, Chapter 29:13, as the primary concern of all these pieces of legislation is the conservation and preservation of the natural environment.

## Approaches to disaster risk reduction

The main purpose of disaster risk reduction is to initiate and complete response and recovery intervention speedily and effectively. Therefore, approaches to disasters have to be adopted so that disaster risk reduction managers may take action quickly and return to normal activities. This study analyses the disaster continuum model, before discussing the expand-contract model.

### The disaster continuum model

The more traditional approach has been to regard disaster management as a number of phased sequences of action. The disaster continuum model is divided into two phases – the pre-disaster risk reduction phase and the post-disaster recovery phase. The pre-disaster risk reduction phase entails activities and actions that should be undertaken before the occurrence of a disaster, whilst the post-disaster recovery phase is concerned with activities in the aftermath of a disaster.

**Figure 3:**
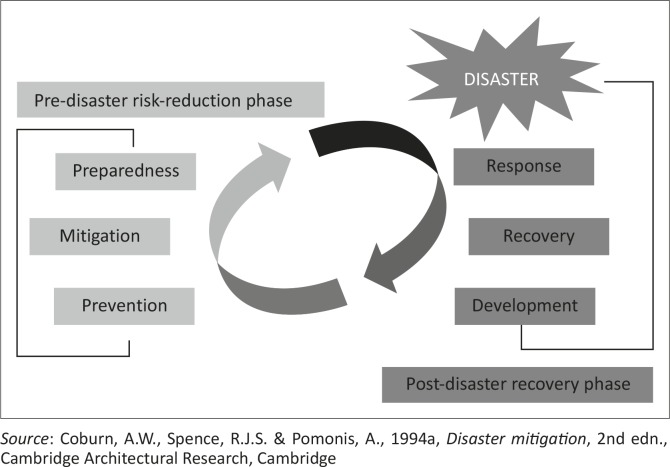
The disaster continuum model.

In the disaster continuum model, disaster risk reduction activities are seen occurring in stages, which follow each other in sequence (Figure 3). Prevention, mitigation and preparedness precede a disaster, whilst response, recovery and development come after the occurrence of a disaster. The major limitation of this model lies in its emphasis on the phased sequences of managing a disaster. It is often observed that the sequences of action may occur simultaneously, hence the modern approach to disaster risk reduction, namely the expand-contract model.

### The expand-contract model

**Figure 4:**
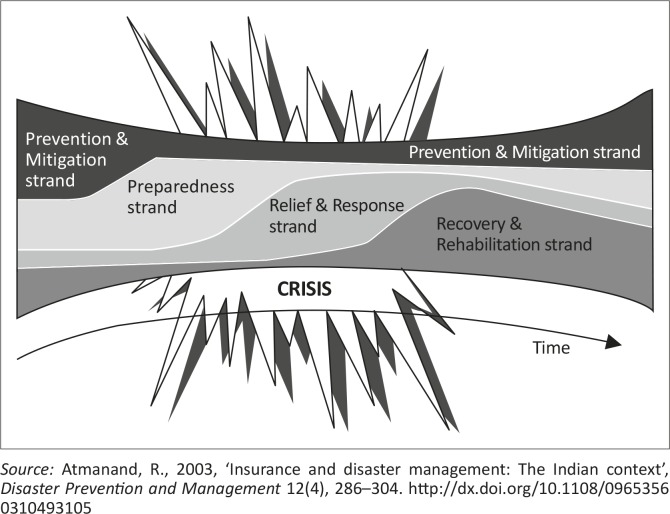
The expand-contract model – a modern approach.

The expand-contract model (Figure 4) provides an alternative view of disaster risk reduction. In this model, disaster risk reduction is seen as a continuous process. According to Atmanand (2003), the expand-contract model presents the phases of disaster risk reduction as a parallel series of activities. The different strands of activities in the expand-contract model continue side by side, expanding or contracting as needed (Asia Disaster Preparedness Centre 2000; Department of Provincial and Local Government 1998). For example, during an anticipated disaster event such as a veld fire, the ‘preparedness’ strand and the ‘prevention and mitigation’ strand will expand to address the pre-disaster needs of the affected community. This model overcomes the major weakness of the disaster continuum model, which regards disasters as managed in a phased sequence.

Despite the existence of these models, disasters are often managed haphazardly. People are often unprepared, which means that disasters trigger haphazard reactions, often resulting in crisis management. A new model is therefore required to address the shortcomings of the expand-contract model, which is not community based.

## Research method and design

### Setting

The study was conducted in Mangwe District of Matabeleland South Province, Zimbabwe. The district shares its boundary with the Republic of Botswana in the west, Matobo District in the east and Bulilima district in the north. The district is made up of 17 wards, namely Izimnyama, Madabe, Tshitshi, Mphoengs, Sanzukwi, Brunapeg, Maninji, Mambale, Bango, Marula, Izimnyama Small scale, Hobodo, Makorokoro, Ngwanyana, Empandeni, Mabuledi and Mkologwe, and three A1 model resettlement areas, namely Watershed, Haygrange and Macingwana. The size of the district is 5722.35 km^2^ and its population is estimated at 66 218 people, based on the 2012 census (Zimstat 2012).

### Design

The study design was mainly qualitative. A qualitative approach was adopted because the research asked for in-depth understanding of human behaviour, feelings, attitudes, perceptions and needs. Qualitative methods are used extensively in analysing these issues (Crang 2002). This type of methodological approach was also chosen because it is very sensitive to human situations, and involves emphatic dialogue with studied subjects.

### Procedure

Questionnaires, interviews and focus-group discussions were used as primary data gathering instruments. The questionnaires were self-administered and were divided into three sections. Section A covered personal details of respondents, whilst Section B dealt with the causes and impacts of veld fires. Finally, Section C analysed disaster risk reduction preparedness, with a focus on current intervention strategies. A total of 20 questionnaires were distributed to members of the DCPU, whilst another 20 questionnaires were administered to members of the Mangwe community.

Interviews provided information that related to people's experiences, opinions, feelings and expectations. Community leaders (chiefs, headmen, village heads and village chairpersons) provided different perceptions towards disaster preparedness for veld fires. A sample of 10 community leaders was interviewed. Through the narration of their views and experiences with this type of disaster, the researcher was able to obtain the required information.

Focus-group discussions were chosen to complement questionnaires and interviews. The researcher brought together 10 members of the District Civil Protection Planning Committee (DCPPC) for the discussion. The group size was kept small deliberately, so that its members did not feel intimidated but could express their opinions freely.

#### Sampling techniques

The sampling methods used in the study are stratified random sampling and snowball sampling. Stratified sampling was the ideal technique, considering that Mangwe District is divided into strata in the form of the 17 wards and 3 resettlements. These wards and resettlements formed 20 strata. Random sampling was then performed by picking one respondent from each stratum, so that each member of the community had an equal chance of being selected. This resulted in a sample of 20 respondents drawn from the whole district. This type of sampling was also used in the selection of community leaders for interview purposes: one community leader was picked from each stratum, resulting in a total of 20 respondents drawn from the district. Another reason for adopting stratified random sampling was to ensure representativeness, even of the smallest subgroups.

Snowballing is a technique that involves research respondents finding other potential respondents. This technique was chosen because it is less time consuming and less costly. The Mangwe District Administrator, who is the chair of the Mangwe DCPPC, was approached. This office then identified other heads of departments and organisations that form part of the Mangwe DCPU. These heads of departments then identified their staff members, who are also members of the DCPU. The heads of departments also identified members of the DCPU who are technical persons. Every identified group had to participate in the study.

## Results

This section presents data collected from the field. The section identifies the major causes of veld fires in Mangwe District, their impact on the community and the current interventions in place. Lastly, other possible intervention strategies are recommended for adoption by the relevant authorities.

### Major causes of veld fires in Mangwe District

**Figure 5:**
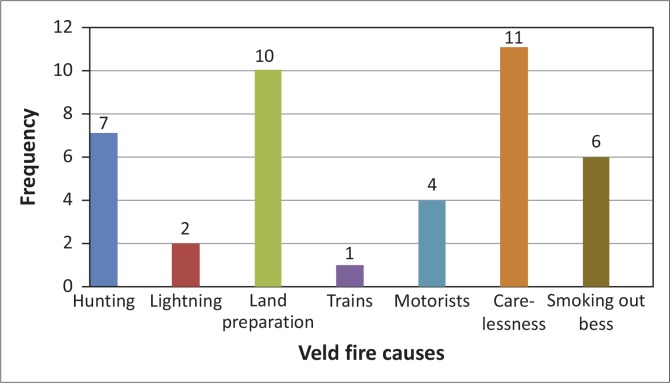
Major causes of veld fires in Mangwe District.

Most veld fires in Mangwe District are human induced. The veld fires occur as a result of human activities such as burning during land preparation for the coming farming season, hunting, smoking out bees, fires kindled by motorists resting along highways, and human carelessness (Figure 5). Human carelessness in the handling of fire (indicated by 11 DCPU members) and burning during land preparation (indicated by 10 respondents) were found to be the two major causes of veld fires. Hunting for wild animals (7 respondents), smoking out bees (6 respondents) were also indicated as causes. Motorists, lightning and sparks produced by trains were found to be minor causes (Figure 5). Lightning is indicated as the only natural factor contributing to veld fires in the district.

These results are similar to the results from previous studies that indicate that most veld fires are human induced. According to Nkomo and Sassi (2009), veld fires outbreaks in southern Africa have resulted mainly from human activities, and natural veld fires have been rare. Mkhwananzi (2007) established that human beings are responsible for 95% of all forest and veld fires.

### Prevalence of veld fires in Mangwe District

Veld fires in Mangwe District usually occur from July to November (Table 2). Of the 20 respondents, 5 (12.5%) mentioned that veld fires occur most in July, 10 (25%) indicated August, 11 (27.5%) indicated September, 11 (27.5%) indicated October, and 3 (7.5%) indicated November. Veld fires in Mangwe District are most prevalent during the months of August, September and October (Table 2). This means that the impact of veld fires is felt most during these months and disaster risk reduction measures should be heightened during this period. Less burning, veld fire awareness campaigns and information on fire danger rating should be focused on during this period.

**Table 2:** Months in which veld fires are most prevalent.

**Month**	**Frequency**	**Percentage (%)**
January	0	0
February	0	0
March	0	0
April	0	0
May	0	0
June	0	0
July	5	12.5
August	10	25
September	11	27.5
October	11	27.5
November	3	7.5
December	0	0
**Total**	**40**	**100**

### Impact of uncontrolled veld fires on communities

The impact of uncontrolled veld fires on communities may be investigated best by analysing its effects on both human livelihoods and the natural environment.

#### The impact of veld fires on livelihoods

**Figure 6:**
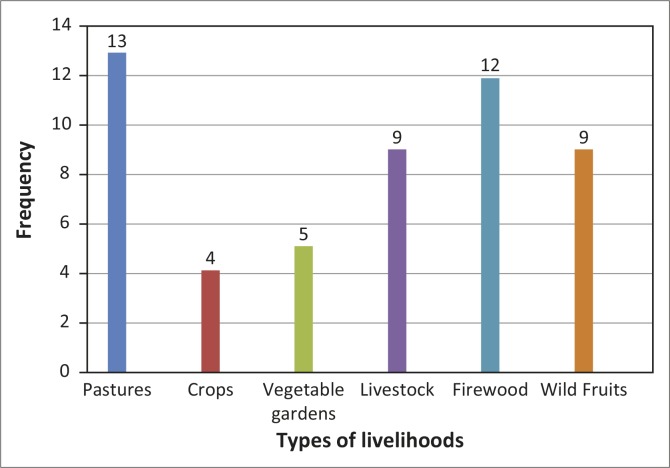
Livelihoods most affected by veld fires.

The types of livelihoods mostly affected by veld fires in Mangwe District are grazing pastures and firewood (Figure 6). Of the 20 respondents, 13 (65%) indicated that pastures are the most affected, whilst 12 (60%) felt that firewood is the most affected. Nine respondents (45%) each indicated wild fruit and livestock, meaning that they are probably affected equally. Crops (five respondents, 25%) and vegetable gardens (four respondents, 20%) have been the least affected livelihoods in the district (Figure 6). These statistics suggest that the communities have employed more effective disaster risk reduction measures to manage the effects of veld fires on crops and vegetables than on other types of livelihoods. It seems the measures taken for livestock have not been effective, although these form part of the most important livelihoods assets. The reason may be that livestock move from one place to another, thereby increasing their vulnerability to veld fires.

#### The impact of veld fires on the natural environment

**Figure 7:**
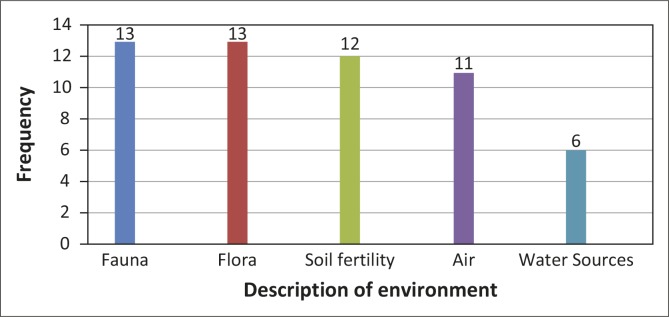
Type of natural environment mostly affected by veld fires.

Veld fires have a devastating effect on the natural environment in Mangwe District. The fauna and flora have been the most affected (Figure 7). Thirteen (65%) respondents showed that fauna have been the most affected, whilst another 13 (65%) indicated flora. Veld fires also destroy soil fertility, according to 12 (60%) respondents. Eleven (55%) respondents felt that veld fires also result in air pollution, whilst six (30%) mentioned that veld fires result in the pollution of water sources. The latter result may imply that veld fires have less adverse impact on the water sources compared to other aspects of the natural environment. The reason may be that the community attaches less value on clean water than on the fauna, flora and soil fertility.

### The Mangwe District Civil Protection Unit and roles of members

A coordinated disaster preparedness and response system is an essential component of any disaster risk reduction plan (Twigg 2007). This study reveals the structure of the Mangwe DCPU, which consists of different government departments, both at central and local government levels, and NGOs. Analysis of the strengths and weaknesses of the members is also highlighted.

The Mangwe DCPU is made up of government departments, including the District Administrator's office, Zimbabwe Republic Police (ZRP), Zimbabwe Prison and Correctional Services, Zimbabwe National Army, Department of Social Welfare, Forestry Commission, EMA, Department of Health and the Central Mechanical Equipment Department. The two local authorities, Plumtree Town Council and Mangwe Rural District Council, are also part of the Mangwe DCPU. NGO members in the unit are the Red Cross Society, International Organisation for Migration, World Vision and Save the Children Zimbabwe, all with different roles.

The District Administrator (DA) chairs the DCPPC on the strength that the DA is the most senior civil servant in the district (Bongo *et al*. 2013; Chikoto & Sadiq 2012). However, the DA's day-to-day duties do not entail disaster risk reduction, therefore the DA may be found wanting in leading a committee on disaster issues. The Department of Social Welfare is mandated with the registration of all NGOs and civic organisations operating in the country. It also monitors their activities, to ensure compliance with the relevant procedures and code of conduct. The strength of members from this department is that they have experience working with disaster-afflicted communities. However, they are more active in the post-disaster recovery phase, that is, during relief, rehabilitation and reconstruction stages of the disaster risk reduction cycle. The ZRP is the law enforcement agency. They enforce statutes such as the *Environmental Management Act* and the *Parks and Wildlife Act*, which are concerned with the preservation and conservation of the natural environment. During disasters, they use their expertise mostly in carrying out search and rescue activities. They search for disaster victims, evacuate them from the scene and render first aid to the injured. The police also carry out campaigns on the dangers of disasters, through their Community Relations Liaison Officers. However, this department does not have a strong resource base. They have scarce resources, such as rescue equipment and vehicles for easy mobility. The Zimbabwe Defence Forces, which consists of the Zimbabwe National Army and the Air Force of Zimbabwe, also play an important role in the committee. Chikoto and Sadiq (2012) observe that these have more resources available and are also responsible for search and rescue activities. However, they are used to the command and control approach, which may not sit well with civilian members of the committee (Mitchell 2003).

The role of the Forestry Commission is to oversee the protection of private forests, trees and forest produce, and to regulate and control the burning of vegetation (Government of Zimbabwe 2002c). The Commission's strength is in providing public education on the dangers of uncontrolled burning, as well as on intervention strategies to be adopted to minimise the damage caused by fires. It also provides the necessary expertise on the construction of standard fireguards to mitigate the impact of veld fires. The local authority is represented by the Plumtree Town Council and the Mangwe Rural District Council in the DCPU. The Town Council provides fire and ambulance services to Plumtree town and the Mangwe District communities in case of emergency. For disasters such as veld fires, the Town Council's fire brigade is a vital part of the DCPU. Its members are well placed to lead disaster risk reduction teams, as they are the only institution in the DCPU with personnel who possess relevant qualifications to manage veld fires. In fact, fire management is part of their core business. Their major weakness is that their expertise is mainly focused on fire and ambulance services. Therefore, they may find it difficult to deal with other types of disasters, such as drought and floods. The Rural District Council is the custodian of all natural resources in Mangwe District. This justifies their inclusion in the DCPU as their area of jurisdiction is affected by veld fires. Their other role is to enforce legislation pertaining to the conservation and protection of the environment; however, this is where their weakness lies: they do not have the capacity to enforce.

The EMA is the environmental watchdog. It has a mandate to oversee the preservation and conservation of the environment so as to ensure that people enjoy a clean environment of high quality. The EMA has launched some campaigns among the communities in the district. Its personnel also ensure that there is compliance with environmental legislation. However, just like the local authorities, the EMA also has no capacity to enforce the laws because its personnel are not trained to do so. Anyone breaking the law under the *Environmental Management Act* is handed over to the police. The Ministry of Health is represented by the local hospital in the DCPU. In the aftermath of veld fires, people who have been injured are treated by the medical personnel either at the disaster scene or at the nearest hospital. However, medical personnel are usually reactive. They are more involved in the post-disaster recovery phase than in the pre-disaster risk reduction phase.

The Central Mechanical Equipment Department is the transport section of the government of Zimbabwe. Their inclusion in the Mangwe DCPU is justifiable, as the need for mobility characterises almost all disasters. During disasters such as veld fires, relocation of the affected communities and livestock usually takes place. Transport of both human beings and their property to relocate the affected communities to safer zones is therefore a necessity that cannot be dispensed with. This Department also provides transport to injured victims should the ambulance services be overwhelmed. NGOs in Mangwe District include the Red Cross Society, the IOM and Save the Children Zimbabwe. All these are a vital component of the institutional framework, as they can assist with regard to resource mobilisation. Usually, they possess the necessary capacity to raise resources because of their strong donor base. Their valuable contribution to disaster preparedness is usually through the provision of fundamental basic human needs such as shelter, medical supplies, food items and water soon after the occurrence of a disaster. In the pre-disaster risk reduction phase, they provide public education and awareness campaigns.

## Mangwe District Civil Protection Unit level of disaster preparedness

An analysis of the disaster risk reduction capacity of the DCPUs in managing veld fires is the crux of the matter in this study.

### Work experience of District Civil Protection Unit personnel

Five members of the DCPU (25% of the 20 respondents) have less than 1 year's experience as members (Table 3). This lack of experience means that the DCPU cannot effectively deal with disasters in the district. Seven (35%) respondents have been in the DCPU for more than 1 year, but less than 5 years. There is need for people with more than 5 years’ experience in the unit so that they would be able to effectively deal with any form of disasters, including veld fires. Three (15%) respondents have served in the unit for more than 6 years, but less than 10 years. This number is very small, considering that it is less than half of the total respondents. Only five (25%) respondents have been dealing with disasters for more than 10 years (Table 3). The implication of these results is that most members of the Mangwe DCPU are lacking disaster risk reduction experience to manage disasters, let alone veld fires. This is a huge setback to development, as disasters would continue to occur in the face of this non-resilient community.

**Table 3:** Experience of District Civil Protection Unit members.

**Experience**	**Frequency**	**Percentage (%)**
Less than 1 year	5	25
1–5 years	7	35
6–10 years	3	15
More than 10 years	5	25

### Effectiveness of current intervention strategies

The DCPU has been managing veld fires in the district through several intervention strategies. Different possible strategies were listed in the questionnaire, so that DCPU members could indicate the ones they were using (Table 4).

**Table 4:** Intervention strategies used by the Mangwe District Civil Protection Unit.

**Current strategy**	**Description**	**Purpose**	**Responsible department**
Firebreaks or fireguard	A strip of land where vegetation has been removed or modified	To reduce the spread and intensity of fire so that damage is minimised	Forestry Commission
Public education or campaigns	Education about veld fires given to community members in order to improve their knowledge	To improve community veld fire awareness	EMA, police, Forestry Commission
Emergency drills	Rehearsals of actions and procedures to be followed in the event of a veld fire outbreak	To improve disaster risk reduction preparedness	All members of DCPU
Law enforcement	Ensuring that relevant Statutes that protect the environment are being followed	Entails arresting the offenders and causing them to pay the gazetted fines	Police, EMA and Forestry Commission officials

EMA, Environmental Management Agency; DCPU, District Civil Protection Unit.

Fireguards, public education, emergency drills and law enforcement were the strategies commonly used by the DCPU to manage veld fires (Table 4). Apart from fireguards, strategies such as enforcement, public education, campaigns and emergency drills were not being used effectively, although they are good interventions. As for enforcement, there is little that the DCPU can do as the non-deterrent fines that offenders are made to pay are determined by the legislature. However, the DCPU is falling short with regard to public education, campaigns and emergency drills. Although the DCPU are providing public education and launching campaigns in the communities, it is on a small scale and infrequently. Only the EMA and ZRP are running some campaigns and providing public education about the dangers of veld fires. However, they do this to fulfil their organisations’ routine mandates, and not in their capacities as members of the DCPU. The DCPU also do not perform rehearsals or drills regularly; according to some respondents, rehearsals or drills are performed once a year. All in all, these strategies have become ineffective because they are not being properly implemented.

### Involvement of beneficiaries

It is important to involve beneficiaries in development initiatives, including disaster risk reduction programmes, in order to enhance programme ownership. Field data from questionnaire respondents, collected from members of the community, revealed that only the EMA and the ZRP are working with communities, as they are running some campaigns and providing public education about the dangers of veld fires.

### Challenges the District Civil Protection Units face in managing veld fires

As indicated by members of the Mangwe DCPU, the DCPU is faced with certain challenges which hinder its risk reduction capacity and readiness to tackle veld fires. Nine respondents out of 20 indicated lack of resources, such as firefighting equipment, funds and transport. Poor coordination amongst the departments that form the DCPU was also cited as one of the challenges affecting disaster risk reduction capacity. Seven respondents felt that good coordination amongst the departments was necessary, as the task entails dealing with emergencies. Six respondents were of the view that lack of properly trained personnel to manage veld fires was another hindrance to disaster preparedness. Inefficiency of the CPPC was also mentioned by five respondents as one of the reasons why the Mangwe DCPU is less prepared to deal with veld fires and other disasters.

## Ethical considerations

Research ethics were observed in carrying out the study. This was meant to improve participation by the respondents. A university clearance, permitting the researcher to conduct study, was obtained and made available to all respondents.

### Potential benefits and hazards

The study was carried out so that the government and its stakeholders, including members of the Mangwe District community, would benefit from the new intervention strategies. There were no risks or harm associated with the study. As such, participants were assured of no physical or psychological harm to anyone. They were also assured that any information gained from them, would be treated with a high level of confidentiality, and that it would be used solely for the purposes of this study.

### Recruitment procedures

Participation in this study was voluntary and none of the respondents was forced to provide information. The respondents were advised that they were entitled to withdraw their consent at any stage of the study if they wished to do so.

### Informed consent

Before the study was conducted, the researcher ensured that all the respondents had consented to participate. Those who had indicated their willingness to participate had their consent checked before they supplied information necessary for the study. This is important and was meant to ensure that their consent had no reservations, but that it was real and not unduly influenced.

### Data protection

Data collected from the respondents was treated with the utmost confidentiality. The researcher ensured that such data was carefully handled, and that there was no unauthorised access to it. The data was used only for the purposes of this study.

## Trustworthiness

### Reliability

The issue of reliability becomes relevant and is of paramount importance if the study is to yield the same result on repeated trials. In this study, questionnaires and interview guides were structured in such a way that they only collect the relevant data. The questionnaires were structured in a way that they were easy to complete, whilst the interview guides contained simple questions soliciting only the required information. Focus-group discussions were designed for information from members of the DCPPC only.

### Validity

Validity concerns the strength of conclusions, inferences or propositions. It is concerned with the soundness and effectiveness of measuring instruments (Leedy 1993). Before the questionnaires and interview guides were used, they were validated through measuring what they intended to measure. These instruments were administered through a pilot survey in order to test them and they succeeded in collecting the necessary data.

## Discussion

### Outline of the results

The main causes of veld fires include hunting, burning during land preparation, motorists, human carelessness, and smoking out bees. Veld fires impact negatively on both people's livelihoods and the natural environment. The livelihoods most affected are grazing pastures and firewood, whilst the impact on the natural environment includes fauna and flora. The DCPU uses various intervention strategies, chief amongst them fireguards, public education, emergency drills and law enforcement. Apart from fireguards, other strategies seem not to be very effective. These strategies need to be complemented by regular training and workshops on veld fire management; the creation of fire protection associations; regular campaigns and rehearsals of emergency drills; and the inclusion of community leaders in the DCPUs. The DCPU is faced with numerous challenges in implementing these strategies. These include lack of resources, such as firefighting equipment, funds and transport. Lack of coordination amongst the departments that form the DCPU is another challenge. There is also a lack of properly trained and experienced personnel to manage veld fires.

### Practical implications

This study provides a new approach to and new strategies for managing veld fires. The main strategies are regular training and workshops on veld fire management; the creation of fire protection associations; regular campaigns and rehearsals of emergency drills; and the inclusion of community leaders, including Ward Development Committees (WARDCOs) and Village Development Committees (VIDCOs), in DCPUs. These strategies inform policy and practice. They can be used by the government and other stakeholders in managing veld fires.

### Towards a community-based disaster risk reduction model

Lack of community involvement in disaster risk reduction programmes in Mangwe District has been observed in this study. Even the DCPU does not incorporate members of the community in its structures. As such, the DCPU is failing to effectively deal with local disasters. The study proposes a new model for improved disaster risk reduction – the community-based disaster risk reduction model. The model encourages community involvement as a pillar for managing disasters. The new model can be used by the DCPU in Mangwe District in particular, other disaster risk reduction practitioners, and academia in solving disaster issues in general.

**Figure 8:**
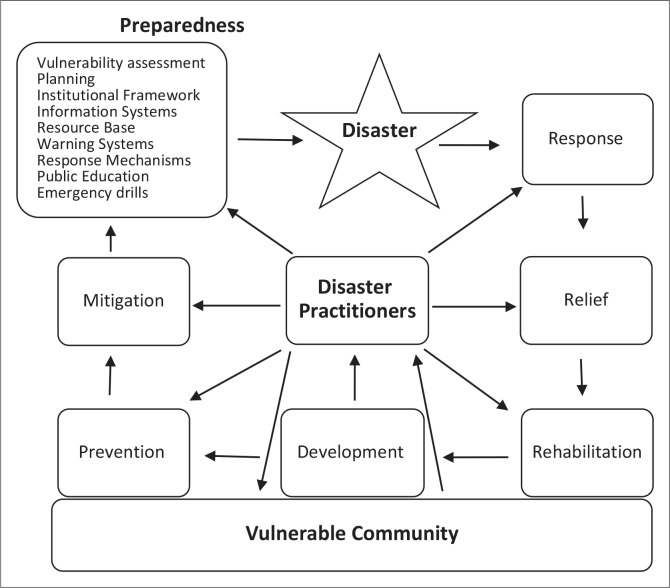
Community-based disaster risk reduction model.

The concept of the community-based disaster risk reduction model (Figure 8) is that, as disaster risk reduction activities focus on benefiting the communities, the local community should form part of the unit responsible for managing local disasters. As preparedness occupies a large part of disaster risk reduction activities, it is indicated by a larger box (see Figure 8). Preparedness follows all elements of the preparedness plan. Arrows indicate direction flow of risk reduction activities. During the response phase, practitioners will consult the preparedness plan to see whether they still have enough resources. They will also share information and assess further vulnerabilities, amongst other things, in the affected community. Thereafter, relief, rehabilitation, development, prevention and mitigation activities will follow. According to this model, the vulnerable community is located at the base of the model, to emphasise that they are the anchor of all disaster risk reduction activities. Disaster practitioners, being the driving force of all activities, are located at the centre of the model. They closely work with communities, who have first-hand information concerning their vulnerabilities. This is one of the advantages of this approach over other models for managing disasters.

### Limitations of the study

Some major limitations were encountered during the study which threatened the smooth progress of the research and reliability of the results. Resistance was encountered from some respondents, who seemed unwilling to release information. Another major limitation was the large size of the study area to be covered.

To ensure reliability of the results, the whole purpose of the research was explained to the respondents in order to increase their cooperation. Confidentiality of their responses was also guaranteed. The researcher also stayed within the community in order to cover the whole study area. It is suggested that future research should provide incentives for respondents, so that participation is encouraged.

### Recommendations

It is recommended that disaster risk reduction practitioners and academia adopt the community-based disaster risk reduction model. Community leaders (chiefs, headmen, WARDCOs and VIDCOs) and members of the communities should be part of DCPU. They should spearhead disaster risk reduction programmes local to their communities. Regular training and workshops on veld fire management should be held with members of the DCPU. The creation of fire protection associations is also recommended. More mature personnel, with more than 5 years’ work experience, should be seconded to the DCPU. Regular campaigns and rehearsal of emergency drills should be undertaken by the DCPUs. Competitions and incentives with regard to veld fire management should be introduced. This would ensure improved preparedness by DCPUs and communities. Vigorous public education and campaigns on the erection of fireguards around homes, cattle pens, crop fields and vegetable gardens in order to limit the spread of veld fires should be undertaken. Lastly, stiffer penalties should be imposed on those who carelessly or deliberately cause veld fires.

## Conclusion

Most veld fires in Mangwe District are caused by human activities, including burning during land preparation, hunting, smoking out bees, motorists resting along highways and general human carelessness. This calls for regulated human behaviour so that this problem does not continue to negatively affect communities. Burning during land preparation and human carelessness are the two major contributors to the veld fires. Controlled burning is a prerequisite, so that unnecessary losses of property, destruction of livelihoods and injury to humans are avoided. Through the destruction of livelihoods such as crops and wild fruits, veld fires may trigger another disaster in the form of drought. As veld fires are at their peak during September and October, it calls for the vigorous implementation of disaster risk reduction strategies during the period. Livestock usually suffer in the aftermath of veld fires, as pastures are destroyed and food is scarce. The natural environment affected by veld fires encompasses fauna and flora, soil fertility, air and water sources. This leads to depleted wildlife and forest reserves. Polluted air and contaminated water are detrimental to human health. The DCPU is less prepared to deal with veld fires and other disasters because the majority of their personnel have limited experience. This means that the problem of veld fires would be a permanent feature in Mangwe District, unless more experienced and competent members are brought into the unit. Only the EMA and the ZRP are running community campaigns on veld fires, and these campaigns have been few. An intensification of such campaigns is called for, so that public education spreads to the entire district. Workshops on veld fire management are rarely held in the district, whilst lack of financial resources, modern equipment and transport has affected operations. Future research should focus on building disaster risk reduction capacities of rural communities for managing local disasters.
